# Open-Flap and Flapless Er, Cr:YSGG Laser-Assisted Esthetic Crown Lengthening: A Comparative Study of Patient Perceptions

**DOI:** 10.1155/ijod/1776661

**Published:** 2025-08-25

**Authors:** Walid Altayeb, Josep Arnabat-Dominguez, Sam B. Low, Ahmed Abdullah, Georgios E. Romanos

**Affiliations:** ^1^Università Cattolica del Sacro Cuore di Roma, Rome, Italy; ^2^Department of Odontoestomatology, Faculty of Medicine and Health Sciences, Dental School, University of Barcelona, Barcelona, Spain; ^3^Department of Periodontology, College of Dentistry, University of Florida, Gainesville, Florida, USA; ^4^Graduate School, Abertay University, Dundee, UK; ^5^Department of Periodontology, School of Dental Medicine, Stony Brook University, Stony Brook, New York, USA

**Keywords:** altered passive eruption, crown lengthening, erbium, gingivectomy, minimally invasive, ostectomy

## Abstract

**Objectives:** This study investigated patients' perceptions of laser-assisted esthetic crown lengthening (ECL) for treating “gummy smiles” using either an open-flap (OF) or flapless (FL) technique.

**Materials and Methods:** This study included 36 healthy patients with altered passive eruption who underwent ECL surgery and were randomly divided into two equal groups: OF and FL. Gingivectomy and ostectomy were performed using an Er, Cr:YSGG laser in both groups. Patients' perceptions of postsurgical morbidity were assessed using a 5-point self-administered Likert scale. The participants provided ratings on their experiences with anxiety, pain, swelling, bleeding, discomfort, tooth sensitivity, and use of pain medication after 7 days. Nine months after surgery, another survey was conducted to assess the overall satisfaction.

**Results:** Patients reported less anxiety, postoperative pain, swelling, bleeding, discomfort, sensitivity, and need for pain medication after the FL than the OF surgery procedure. Both groups reported high satisfaction with the final cosmetic outcome after 9 months, although patients who underwent FL surgery tended to report even greater satisfaction. The FL procedure was also significantly faster, taking approximately 15 min less than OF surgery.

**Conclusions:** The FL laser-assisted ECL technique led to a more positive recovery and better patient experiences than the OF technique.

**Clinical Relevance:** This study suggests that laser-assisted FL ECL may be a more patient-friendly and efficient procedure compared to traditional OF surgery with similar esthetic results. This could be important for dentists looking to offer their patients a less invasive and potentially more comfortable option.

## 1. Introduction

Esthetic crown lengthening (ECL) surgery may be considered an appropriate procedure to address the excessive gingival display associated with altered passive eruption and to achieve a more balanced and symmetrical smile [1, 2]. Several factors, such as the width of the keratinized gingiva, position of the gingival margins, buccal alveolar crest location, mucogingival junction location, and the potential need for restorative therapy, contribute to the determination of the ECL approach [3, 4]. Two options were proposed for treating periodontal cases that require ostectomy to create enough room for supra crestal gingival tissues: apically repositioned full-thickness flap and gingivectomy followed by flapless (FL) osteotomy [5].

Literature suggests that various instruments can be used in ECL to obtain a more physiological gingival contour, including a scalpel, electrosurgery, or laser [4]. The application of lasers in periodontal surgery has expanded significantly, especially over the last two decades. This growth is primarily fueled by the pursuit of minimally invasive dentistry and the unique advantages lasers offer: precise, efficient, and safe ablation of periodontal tissues. Lasers also provide effective disinfection and hemostasis, making them a preferred tool in periodontology [6]. The Er, Cr:YSGG 2780-nm wavelength is well absorbed by water, the major chromophore in soft and hard tissues. The ability of the Er, Cr:YSGG laser to ablate both hard and soft tissues with excellent surgical precision and minimal collateral effects creates an opportunity for minimally invasive flaps and FL ECL, resulting in decreased tissue damage, minimized bleeding, and reduced inflammation and postoperative discomfort, thus, enhancing healing [7, 8]. Clinical case reports suggest that laser-assisted FL ECL might allow faster and uneventful wound healing and eliminate irregular tissue positioning due to tension that occurs in the tissue when the flap is reflected and sutured, in addition to the overall positive experience for the patient [9, 10].

Patient satisfaction is an essential measure in healthcare that indicates how patients perceive the quality of care they receive in relation to their expectations. Moreover, it provides insights into the overall effectiveness of the treatment and facilitates comparisons between various treatment options [11]. The purpose of this study was to compare how patients perceive the morbidity and esthetic satisfaction of laser-assisted open flap (OF) and FL ECL procedures for treating altered passive eruptions over a 9-month healing period. In a previous publication [12], we presented the clinical results of this study, which analyzed the positional changes in periodontal tissues after ECL. Specifically, we evaluated the supracrestal gingival tissue dimensions, biological width, and stability of crown height by comparing these parameters between the OF and FL groups.

## 2. Materials and Methods

### 2.1. Study Sample

This study enrolled 36 healthy patients (14 males and 22 females) aged 22–45 years between August 2014 and March 2019. All patients presented with excessive gingival display caused by altered passive eruption of the six anterior maxillary teeth. These teeth also exhibited short clinical crowns and discrepancies in the gingival margin height. Following informed consent, the participants were randomly allocated into two groups (OF and FL), with 18 patients each. Adaptive randomization was employed to minimize imbalance between treatment groups by dynamically adjusting allocation probabilities throughout the study. A stringent enrollment criterion for the FL group was the maintenance of at least 2 mm of attached keratinized gingiva (AKG) on all six anterior maxillary teeth following gingivectomy, as dictated by the predetermined esthetic treatment plan. The initial subject was allocated via simple randomization (coin toss). Subsequent subjects were then randomly assigned to either the OF or FL group, each comprising 18 patients, with their allocation informed by the responses of previously enrolled participants. The patients were informed of the nature and potential risks of the proposed surgical procedures and reviewed and signed an informed consent form. The study was conducted in accordance with the principles of the Declaration of Helsinki.

### 2.2. Inclusion and Exclusion Criteria

#### 2.2.1. Inclusion Criteria

The inclusion criteria were as follows: (1) Patients with an excessive gingival display of at least 3 mm; (2) gingival overlap >19% of the anatomical crown height; (3) age >22 years; (4) at least six anterior maxillary teeth requiring ECL; and (5) healthy periodontal status, indicated by a full-mouth plaque index and bleeding on probing index scores of <15%.

#### 2.2.2. Exclusion Criteria

The exclusion criteria were as follows: (1) treatment sites with probing depth ≥3 mm; (2) planned restorative procedures involving apical repositioning of the incisal edge, potentially affecting the mock-up fit at follow-up appointments; (3) pregnant or lactating patients; (4) patients with a history of smoking; (5) patients requiring preprocedural antibiotic prophylaxis; (6) patients with a history of mucogingival surgery in the intended treatment area; (7) systemic conditions that may impair tissue healing (e.g., uncontrolled diabetes and autoimmune diseases); (8) patients undergoing active orthodontic treatment.

### 2.3. ECL Surgery

Both groups underwent crown lengthening using Er, Cr:YSGG 2780-nm (iPlus Waterlase, Biolase, USA)^1^. The OF group was treated with an apically positioned flap and osseous recontouring was performed. The FL group was treated with a FL technique that included gingivectomy and osseous recontouring. These procedures were conducted as described in the previous publication [12].

### 2.4. Patients' Perceptions and Esthetic Satisfaction

Patients' perceptions of postsurgical morbidity were assessed using a self-administered questionnaire. The study used a 5-point Likert scale to measure anxiety immediately after surgery, with 1 indicating “minimal anxiety” and 5 indicating “extreme anxiety.” One week after surgery, the patients were asked to rate their levels of pain, swelling, bleeding, and tooth sensitivity on a 5-point Likert scale. The scale ranged from 1 (“not at all”) to 5 (“very severe”). To assess the difference between the patients' preoperative expectations and their actual experiences concerning the complexity of the procedure, the same 5-point Likert scale was used. The scale ranged from 1 (“much worse more than expected”) to 5 (“much better than expected”). Patients were also monitored and their anti-inflammatory medication intake (400 mg ibuprofen) was recorded throughout the first week.

Nine months after surgery, a separate questionnaire was used to evaluate the patients' overall satisfaction and esthetic appearance. This questionnaire also addressed specific aspects of patient experience, including satisfaction with the smile, gingival display, and the best and worst aspects of the procedure, using a 5-point Likert scale, with 1 indicating “Very dissatisfied” and 5 indicating “very satisfied.”

The duration of the surgical procedure was carefully recorded to guarantee precise evaluation of both methods. We used a standardized methodology, starting the timer as soon as the laser was applied and stopped immediately when the patient left the dental chair.

### 2.5. Statistical Study

In this study, we used the Mann–Whitney *U* test, a nonparametric test, to assess the differences between the two independent groups. The *t*-test was used for parametric data that exhibited a normal distribution. A significance level of 0.05 was used for the Mann–Whitney *U* test and *t*-test. Relationships between variables were analyzed using Spearman's rank correlation coefficient, with a 0.01 significance level applied to the test. Data analysis was performed using computer software (SPSS v.17.0, IBM, Chicago, IL).

## 3. Results

Statistical analyses were performed on 216 teeth (108 in each group) from 36 patients. Patients' perceptions of morbidity and esthetic appearance are presented in Tables 1 and 2.

**Figure 1:**
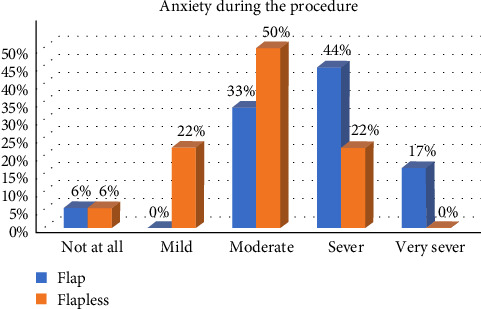
The difference in the levels of patient anxiety immediately after surgery between the two groups.

The results showed a significant difference in the levels of patient anxiety between the two crown-lengthening methods (Figure 1). Within the OF group, 61% of the patients reported feeling anxious during the procedure, whereas only 22% of the patients in the FL group reported similar levels of anxiety (*p*=0.009). The average operative time for surgery in the OF group was 77.8 ± 7.46 min compared to 62.7 ± 4.87 min in the FL group. The FL technique was significantly faster, with an average time of 15 min lesser than that of the OF technique. (*p*=0.043)

**Figure 2:**
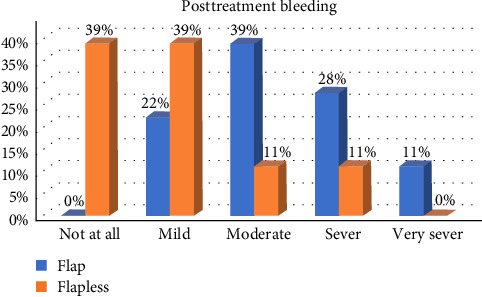
The difference in the levels in postsurgical bleeding between the two groups during the first week.

**Figure 3:**
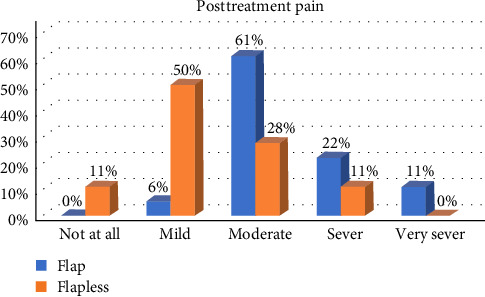
The difference in the levels in postsurgical pain between the two groups during the first week.

**Figure 4:**
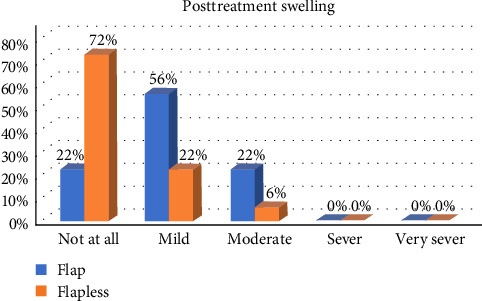
The difference in the levels in postsurgical swelling between the two groups during the first week.

**Figure 5:**
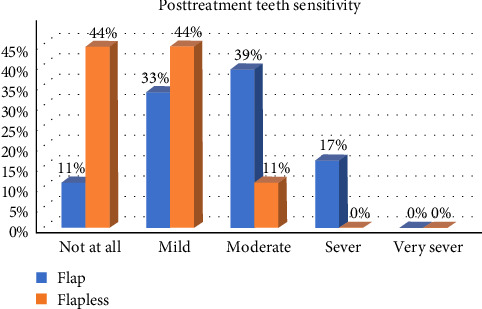
The difference in the levels in postsurgical teeth sensitivity between the two groups during the first week.

In general, postoperative healing was uneventful for the study participants, and the patients reported low levels of morbidity after both surgical procedures. When comparing OF and FL, we detected a significant difference in postsurgical bleeding, postsurgical pain, swelling, and teeth sensitivity during the first week, with OF patients reporting higher scores (*p* < 0.05; Figures 2–5).

Most patients (88%) reported taking anti-inflammatory medications to relieve pain and reduce swelling for up to 3 days after surgery and there was a significant difference between the groups (*p*=0.004). A significantly higher proportion of patients in the OF group (63%) continued taking medication beyond the third day than those in the FL group (25%; *p* < 0.05).

**Figure 6:**
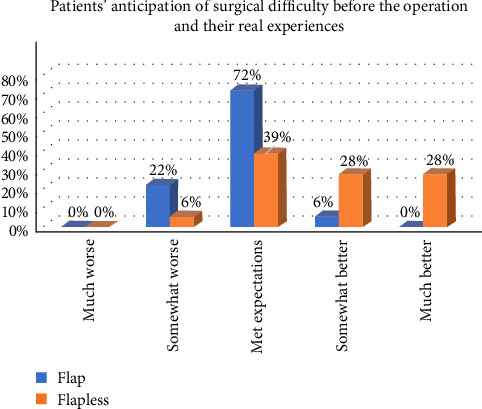
The difference between the patients' preoperative expectations and their actual experience between the two groups concerning the complexity of the procedure.

The findings regarding the difference in patients' anticipation of surgical difficulty before the operation and their real experiences after the operation indicated that 72% of patients with OF and 39% of patients with FL reported a postoperative experience consistent with their initial expectations of surgical difficulty. Additionally, a considerably greater percentage (56%) of FL patients found the surgery to be less challenging than they had anticipated, compared to only 5% of OF patients (*p* < 0.05; Figure 6).

**Figure 7:**
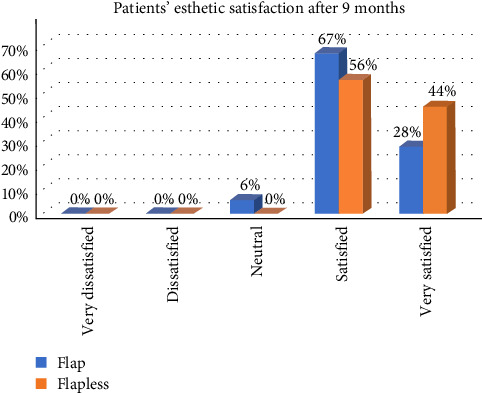
The difference between the patients' overall esthetic satisfaction between the two groups after 9 months.

In the FL group, patients reported higher satisfaction scores with regard to esthetic appearance 1 week after surgery (*p* < 0.05). There were no differences between the groups regarding satisfaction with esthetic appearance after 9 months (*p*=0.234). This study revealed a statistically significant difference in patient satisfaction between the two groups. Considering the entire experience, including surgery, recovery, and final outcome, all patients in the FL group reported a high likelihood of undergoing the procedure again, if needed. In contrast, only 75% of patients in the OF group expressed a similar likelihood; however, there was no significant difference between the two groups (*p*=0.07; Figure 7).

Similarly, a statistically significant difference emerged in willingness to recommend the procedure. All patients in the FL group were willing to recommend this procedure to individuals facing similar issues, in contrast to 89% of the patients in the OF group, who would extend such a recommendation, without a significant difference between the two groups (*p*=0.38). The statement “Accepting to repeat the same treatment if necessary” was inversely correlated to the degree of stress (pain and the length of the surgery) during the procedure (*r* = –0.53, *p* < 0.001).

## 4. Discussion

Providing excellent dental care involves considering the patients' perspectives. Patients' perceptions and satisfaction can affect treatment decisions, and adjustments can be made to increase satisfaction levels [13]. Many studies have shown a strong link between patient satisfaction and adherence to treatment, which is now recognized as a crucial factor in healthcare outcomes, including those in the field of dentistry [14]. Therefore, patient satisfaction has been proposed as a key metric for assessing the overall effectiveness of periodontal treatment, allowing dentists to compare different approaches and identify areas for improvement [15].

This study investigated patients' perceptions of and satisfaction with the final results of laser-assisted ECL with flap and FL techniques using data from a previously published study focusing on surgical techniques and clinical outcomes. Although the methodology and dataset remained consistent, our focus shifted to understanding patient experiences to provide a more comprehensive understanding of laser-assisted ECL and its potential benefits [12].

The Er, Cr:YSGG 2780-nm laser was chosen because it can handle both soft and hard tissues. Precise, efficient, and safe ablation of periodontal tissues without collateral thermal damage were the main reasons for choosing a surgical laser in periodontology [16].

Our study revealed a significant difference in anxiety levels between the two methods of crown lengthening. Individuals who underwent flap surgery reported higher levels of anxiety than those who underwent FL surgery. This finding suggests that the less-invasive nature of the FL technique may lead to a more positive patient experience [17, 18].

Almost all patients had mild-to-moderate postsurgical morbidity after ECL. This result is in good agreement with many studies that have shown that the overall incidence of postsurgical pain is low following periodontal surgery, and the intensity is mild for the majority of patients [15, 19, 20]. Patients who undergo FL surgery have reported lower levels of bleeding on the day after the surgery and reduced levels of pain, swelling, and discomfort in the first week. These results align with the findings of Ribeiro et al. [21] who noted that patients who underwent traditional flap surgery tended to experience higher pain scores than those who underwent the FL approach. This correlation is possibly because the FL technique involves less tissue manipulation and surgical trauma. By minimizing tissue reflection, the FL approach is believed to facilitate quicker healing and more comfortable postoperative recovery for patients [22].

Periodontal flap surgery typically requires greater extension and/or a longer average duration of the procedure, thereby potentially exacerbating anxiety and stress among patients, and consequently leading to heightened levels of discomfort and postoperative pain [23, 24]. Multiple studies have consistently demonstrated that ECL is a safe and minimally morbid procedure [25, 26].

The use of anti-inflammatory medications provided insights into the potential differences in postoperative pain between the two techniques [27]. Although the majority of patients in both groups acknowledged the need for medication during the first 3 days after surgery, a significantly higher percentage of patients in the OF group continued to use medication beyond the third day than those in the FL group. The higher invasiveness of the OF technique, which can result in more extensive surgical trauma and tissue disturbances, may account for the necessity of prolonged pain control [28].

This study revealed a significant difference in how well the patients' preoperative expectations regarding surgical difficulty aligned with their actual postoperative experience. A significantly higher percentage of patients who underwent flap surgery reported that postoperative difficulty matched their preoperative expectations. In contrast, a substantially higher proportion of patients in the FL group found surgery easier than anticipated preoperatively. This may be attributed to the less invasive nature of the FL technique and shorter operation duration, which could have caused patients to underestimate its complexity, resulting in a higher number of patients finding it easier than initially expected [17].

In general, patients reported high levels of satisfaction with the esthetic results related to the size and visibility of the teeth, and gingival display for both surgical procedures after 9 months, with a more positive perception of the FL method. These results agree with the conclusion of Ribeiro et al. [21] who suggested using the FL procedure because it seems to be a feasible, predictable, and time-saving method for the treatment of gummy smiles caused by altered passive eruption relative to flap elevation, which can sometimes induce an intensified bone remodeling process. Using the laser in this study might have influenced the higher satisfaction scores for both methods compared with the results of Ribeiro et al. [21]. In general, patients' perceptions of lasers are positive, and they feel that lasers can make their visit to the dentist less traumatic [29]. Considering the entire experience from surgery to recovery and the final outcome, all patients in the FL group and 75% of the patients in the OF group had a strong likelihood of undergoing the procedure if needed. This is consistent with the findings of Silva et al. [30] who reported that all patients in their study would undergo the procedure again and recommend it to others. Based on limited clinical studies and case reports, lasers offer significant advantages, including less intraoperative trauma and bleeding, shorter operative times, and improved postoperative pain management [6].

Our analysis revealed a significant benefit of the FL technique in terms of surgical efficiency. The FL group demonstrated a notably shorter average time for operations, with up to 20% reduction compared to the OF group. These findings are consistent with those of previous research conducted by Ribeiro et al. [21] who reported a 25% reduction in surgery time when using the FL technique instead of traditional methods. The shorter operative time associated with the FL technique can be attributed to elimination of the steps involved in raising and repositioning the flap. This streamlined approach may benefit dental professionals by improving efficiency and potentially reducing patient anxiety due to a shorter chair time.

## 5. Conclusion

In conclusion, this study explored patients' perceptions of ECL procedures using two surgical techniques: laser-assisted FL and OF. FL surgery resulted in significantly lower anxiety and postoperative pain than OF surgery. Although both techniques achieved successful clinical outcomes, patients reported greater satisfaction with their overall experience, including surgical time and recovery, when undergoing the FL procedure. These findings suggest that FL surgery may be a superior approach to crown lengthening, offering a less invasive and more positive patient experience. By prioritizing patient satisfaction and incorporating patient perspectives, dental professionals can improve the quality of care and achieve superior clinical results.The study's limitations include the absence of blinding, the relatively small sample size, and the single-operator design. These factors may influence the generalizability and objectivity of patient-reported outcomes.

## Figures and Tables

**Table 1:** Patient perceptions regarding morbidity after both surgical procedures.

The self-administered questionnaire	Treatment group		Mann–Whitney *U*	*p*-Value
	OF	FL		
	Mean rank	Mean rank		
Anxiety during the procedure?	22.81	14.19	84.5	0.009*⁣*^*∗*^
Did you experience bleeding during the week following treatment?	24.39	12.61	56	<0.001*⁣*^*∗*^
Did you experience pain during the week following treatment?	23.89	13.11	65	0.001*⁣*^*∗*^
Did you experience swelling during the week following treatment?	23.17	13.83	78	0.004*⁣*^*∗*^
Did you experience sensitive teeth during the week following treatment?	23.56	13.44	71	0.002*⁣*^*∗*^
How was the procedure experience compared to what you thought it would be?	13.44	23.56	71	0.001*⁣*^*∗*^
Discomfort and teeth sensitivity during the week following the surgery	22.61	14.39	−2.53	0.013*⁣*^*∗*^

*⁣* ^*∗*^The mean difference is significant at the 0.05 level.

**Table 2:** Patient perceptions regarding esthetic appearance after both surgical procedures.

The self-administered questionnaire	Treatment group		Mann–Whitney *U*	*p*-Value
	OF	FL		
	Mean rank	Mean rank		
Did you notice a cosmetic change after 1 week?	11.89	25.11	43	<0.001*⁣*^*∗*^
Did treatment meet your expectations after 9 months?	16.72	20.28	130	0.234*⁣*^*∗*^
Would you repeat treatment if necessary?	15.78	21.22	113	0.078
Would you recommend this procedure to someone with a similar problem?	17.28	19.72	140	0.388

*⁣* ^*∗*^The mean difference is significant at the 0.05 level.

## Data Availability

The data that support the findings of this study are available from the corresponding author upon reasonable request.

## Endnotes

^1^Iplus Waterlase, Biolase, Irvine, CA, USA.
